# Reoperative surgery for early- and late-onset prosthetic valve endocarditis: temporal trends and outcomes

**DOI:** 10.1093/icvts/ivaf096

**Published:** 2025-04-11

**Authors:** Pradeep Narayan, Tim Dong, Jeremy Chan, Charles Tan, Tugba Aydin, Shubhra Sinha, Daniel P Fudulu, Gianni D Angelini

**Affiliations:** Cardiac Surgery, Narayana Health, Kolkata, India; Cardiac Surgery, Bristol Heart Institute, Bristol University, Bristol, UK; Cardiac Surgery, Bristol Heart Institute, Bristol University, Bristol, UK; Cardiac Surgery, Bristol Heart Institute, Bristol University, Bristol, UK; Cardiac Surgery, Bristol Heart Institute, Bristol University, Bristol, UK; Cardiac Surgery, Bristol Heart Institute, Bristol University, Bristol, UK; Cardiac Surgery, Bristol Heart Institute, Bristol University, Bristol, UK; Cardiac Surgery, Bristol Heart Institute, Bristol University, Bristol, UK; Cardiac Surgery, Bristol Heart Institute, Bristol University, Bristol, UK

**Keywords:** prosthetic valve endocarditis, aortic valve endocarditis, reoperative aortic valve surgery

## Abstract

**OBJECTIVES:**

Prosthetic valve endocarditis (PVE) remains a serious complication following aortic valve replacement, with varying outcomes based on timing of presentation. This study investigated the relationship between timing of reoperation and outcomes in PVE patients using a nationwide database, while examining temporal trends and identifying mortality risk factors.

**METHODS:**

We analysed 406 patients who underwent reoperative surgery for PVE between 1996 and 2019 across the United Kingdom using the National Institute of Cardiovascular Outcomes Research database. Patients were categorized into early (≤1 year, n = 131) and late (>1 year, n = 275) reoperation groups. Propensity score matching was performed to compare outcomes, and multivariable analysis identified mortality predictors.

**RESULTS:**

The overall incidence of reoperative surgery for PVE increased over the study period, while the proportion of early-onset PVE decreased from 85.7% in 1998 to 20% in 2019. In matched groups (113 pairs), early reoperation cases required longer cardiopulmonary bypass (135 vs 122 min, *P* = 0.005) and cross-clamp times (95 vs 88 min, *P* = 0.011), with extended hospital stays (18.5 vs 14.0 days, *P* = 0.006). Overall mortality was 8.4%, with an early mortality of 10.8% opposed to 6.2% in late cases (*P* = 0.338; Lasso AUC: 0.746). Age emerged as the strongest predictor of mortality (OR: 1.14, 95% CI: 1.05–1.27, *P* = 0.0).

**CONCLUSIONS:**

This nationwide analysis demonstrates improved PVE outcomes compared to historical data, with overall mortality of 8.4%. While early reoperations were associated with more complex procedures and longer hospital stays, mortality rates were statistically similar. Age remains the primary determinant of mortality risk.

## Graphical Abstract

**Figure:**
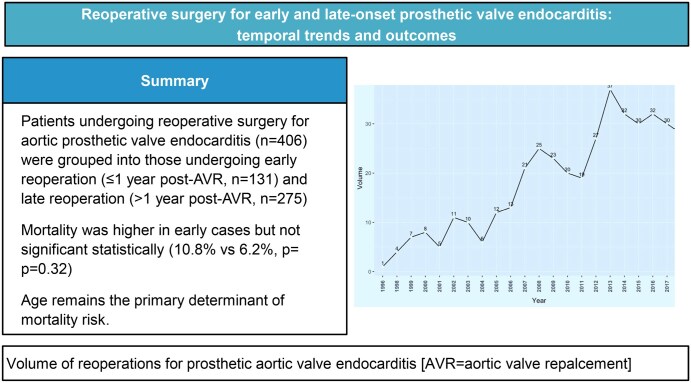

## INTRODUCTION

Prosthetic valve endocarditis (PVE) is a serious complication following aortic valve replacement (AVR), accounting for 10–30% of all infective endocarditis cases [1–3]. This condition occurs in approximately 3 to 6% of patients within 5 years of valve implantation [4]. Despite advances in surgical techniques and medical management, reoperative AVR due to PVE remains a challenging procedure with considerable risks and is associated with significant in-hospital mortality ranging from 12 to 28% [2, 5, 6]. Valve type, bio-prosthetic or mechanical, has not been shown to be associated with a predilection for PVE [7].

Early PVE is predominantly considered a healthcare-associated infective endocarditis and differs significantly from late PVE in terms of causative microorganisms and optimal antibiotic regimens. The definition of early PVE varies, with some studies defining it as occurring within 60 days post-surgery [8], while others extend this period to 6 months [9]. However, the definition recommended by the Task Force for the Management of Infective Endocarditis of the European Society of Cardiology suggests 1-year to be the cut-off for defining early PVE [7]. This extended definition is supported by epidemiological data showing that 71% of healthcare-associated PVE cases occur within the first year following valve implantation, underscoring the critical nature of this early postoperative period [8]. While numerous studies have explored various aspects of PVE, there is a notable paucity of evidence regarding the impact of timing of presentation on patient outcomes. This has been largely understudied and often suffer from small sample sizes and un-matched comparisons [9].

Our study aims to address these critical issues by utilizing a large, nationwide UK propensity-matched dataset to investigate the relationship between timing of presentation and patient outcomes following reoperative AVR due to PVE. We also sought to examine the key risk factors contributing to mortality in patients undergoing re-operations for PVE.

## METHODS

This retrospective study analysed patients undergoing reoperative aortic valve surgery in the United Kingdom from January 1996 to March 2019, using data from the National Institute for Cardiovascular Outcomes Research (NICOR) registry. The NICOR database systematically collects demographic, intraoperative, and outcome data as part of the National Adult Cardiac Surgery Audit. The rate of missing data concerning baseline information within the NICOR registry remained exceedingly low, at 1.7%. Data processing and imputation of missing data were as previously described [10].

The primary aim of the study was to investigate the influence of the timing of presentation on patient outcomes after reoperative AVR due to PVE. Additionally, the study sought to identify specific risk factors associated with mortality following reoperative AVR. Patients were classified into two groups based on the timing of PVE onset: early-onset PVE (≤1 year after the initial operation) and late-onset PVE (>1 year after the initial operation). Patients with native valve endocarditis or those undergoing reoperations necessitated by structural valve degeneration were excluded from the study. We also excluded patients who required concomitant surgery or those requiring more extensive procedures like an aortic patch or aortic root replacement due to aortic root abscesses. To evaluate the temporal trends in PVE presentations, we analysed the annual proportion of cases presenting early (≤1 year after initial valve surgery) versus late (>1 year after initial valve surgery) from 1996 to 2019.

### Statistical analysis

Categorical variables were expressed as counts and proportions, and comparisons were made using either the Pearson's Chi-squared test or Fisher's exact test. Continuous variables were presented as medians and interquartile ranges (IQRs). The binary dependent variable in this analysis was defined to capture the treatment effect, differentiating between early (≤1 year) and late (>1 year) reoperation groups. To assess the treatment effect, propensity score matching was applied. Patients were precisely matched in a 1:1 ratio using the nearest-neighbour method, with a calliper width of 0.1 standard deviations of the logit of the propensity score. Variables included in the comprehensive logistic regression model are enumerated (Table S1). A standardized mean difference greater than 0.10 indicated residual imbalance among variables. Match quality was visually assessed using a Love plot (Fig. S1) and a mirror plot, which depicted the common support area of propensity score values shared between the two groups (Fig. S2). For the matched cohort, the Wilcoxon signed-rank test was employed to compare continuous variables, while the McNemar test was used for categorical variable.

Additionally, a multivariable logistic regression analysis was performed to identify independent predictors of mortality following reoperative AVR due to PVE (Table S2). The analysis utilized the Akaike Information Criterion (AIC) for model selection based on 500 bootstrap datasets, with an alpha level set at 0.05. The variables included in the regression model are provided in Table S2. Odds ratios (OR) along with 95% confidence intervals (CI) were computed for each variable, with statistical significance determined at *P* < 0.05.

### Predictive analysis

The unmatched (*n* = 406) dataset was used for the predictive modelling due to the smaller sampler size of the matched set. The 5 samples with missing outcomes were excluded resulting in 401 samples for predictive modelling. The unmatched (*n* = 401) dataset was first split into training/validation and holdout sets using 70% (281): 30% (120) split. Random stratified 5-fold Grid Search Cross Validation was applied on the training/validation dataset to determine the optimal hyperparameters and validation performance. Feature selection was conducted using different thresholds of absolute coefficient magnitude for removing variables below the thresholds. The Lasso model was then retrained on the training/validation dataset using the optimal settings identified. The model was then evaluated on the hold out set to obtain AUC performance. A Support Vector Machines with Radial Basis Function Kernel model was also be evaluated using the same process as above.

### Ethical considerations

The study received ethical approval from the Health Research Authority (HRA) and Health and Care Research Wales (HCRW). Given that the research involved retrospective analysis of the NICOR database, individual patient consent requirements were waived in accordance with HCRW guidelines (IRAS [Integrated Research Application System] ID: 257758).

## RESULTS

**Figure 1:**
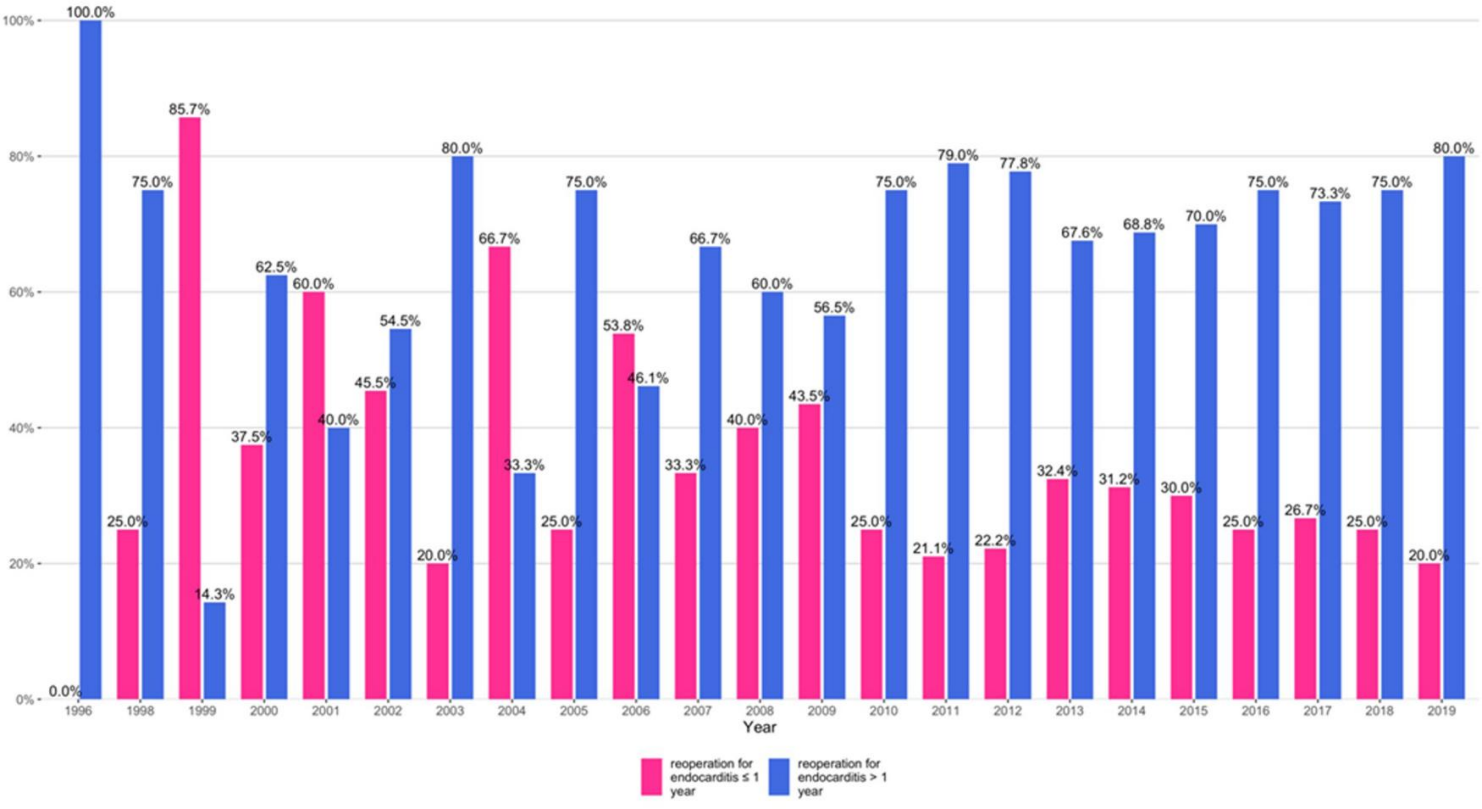
Trend of case volumes presenting early (≤1 year) and late (>1 year) among patients with prosthetic aortic valve endocarditis.

Between 1996 and 2019, a total of 406 patients underwent isolated prosthetic valve reoperation due to endocarditis, with 131 patients needing a reoperation within 1 year (early reoperation group) and 275 needing a reoperation after 1 year of the index surgery (late reoperation group). A consort diagram has been provided (Fig. S3). Early reoperations were most common in the early part of the study with 85.7% (in 1998) which declined to a low 20% in 2003. From 2010 onwards, late reoperations consistently outnumbered early ones with 80% occurring after 1 year and 20% within 1 year in 2019 (Fig. 1).

The groups were comparable in terms of age (69.10 vs 67.50 years; *P* = 0.891) and sex distribution (23.7% vs 20.4% females; *P* = 0.53). Other baseline characteristics, were also comparable between the groups (Table 1). After propensity score matching, 113 patients were included in each group for comparative analysis. Most baseline characteristics were well-balanced and showed similar rates of comorbidities such as diabetes mellitus, hypertension, chronic kidney disease and previous cerebrovascular events (Table 2).

**Table 1:** Unmatched comparison of baseline characteristics

	Duration of reoperation for endocarditis ≤ 1 year (n = 131)	Duration of reoperation for endocarditis > 1 year(n = 275)	*P*-value
Age (years)	69.10 [60.80, 73.00]	67.50 [58.95, 75.05]	0.891
Female	31 (23.7)	56 (20.4)	0.53
NYHA functional class			0.601
NYHA I	31 (23.7)	51 (18.5)	
NYHA II	34 (26.0)	68 (24.7)	
NYHA III	41 (31.3)	96 (34.9)	
NYHA IV	25 (19.1)	60 (21.8)	
CCS grade			0.894
CCS 0	102 (77.9)	205 (74.5)	
CCS 1	16 (12.2)	36 (13.1)	
CCS 2	8 (6.1)	17 (6.2)	
CCS 3	3 (2.3)	10 (3.6)	
CCS 4	2 (1.5)	7 (2.5)	
EuroSCORE II	8.15 [5.53, 15.79]	8.90 [6.05, 16.50]	0.444
Body mass index, kg/m^2^	26.30 [24.04, 29.44]	26.23 [23.36, 29.15]	0.849
Diabetes mellitus			0.343
Non-diabetics	106 (80.9)	237 (86.2)	
On diet control	3 (2.3)	8 (2.9)	
Oral medication	15 (11.5)	23 (8.4)	
Insulin	7 (5.3)	7 (2.5)	
Hypertension	71 (54.2)	154 (56.0)	0.815
Smoking			0.052
Never smoked	52 (39.7)	116 (42.2)	
Former smoker	78 (59.5)	144 (52.4)	
Current smoker	1 (0.8)	15 (5.5)	
Chronic kidney disease	21 (16.0)	28 (10.2)	0.126
Chronic obstructive pulmonary disease	11 (8.4)	31 (11.3)	0.474
Previous myocardial infarction	12 (9.2)	22 (8.0)	0.839
Previous cerebrovascular attack	23 (17.6)	51 (18.5)	0.917
Peripheral vascular disease	10 (7.6)	28 (10.2)	0.521
Preoperative atrial fibrillation	17 (13.0)	40 (14.5)	0.785
Pulmonary hypertension	4 (3.1)	10 (3.6)	0.992
Left ventricular ejection fraction			0.676
Poor < 30%	48 (36.6)	91 (33.1)	
Moderate 30–50%	1 (0.8)	4 (1.5)	
Good ≥ 50%	82 (62.6)	180 (65.5)	
Cardiogenic shock	6 (4.6)	16 (5.8)	0.779
Aortic valve explant: biological valve	102 (77.9)	180 (65.5)	0.015
Pre-op ventilation	3 (2.3)	13 (4.7)	0.364

CCS, Canadian Cardiovascular Society; NYHA: New York Heart Association.

**Table 2:** Propensity-matched comparison of baseline characteristics

	Duration of reoperation for endocarditis ≤ 1 year (n = 113)	Duration of reoperation for endocarditis > 1 year(n = 113)	*P*-value	Unmatched SMD	Matched SMD
Age (years)	69.20 [61.70, 73.10]	69.70 [60.80, 75.40]	0.448	0.04	0.08
Female	27 (23.9)	25 (22.1)	0.874	0.08	−0.04
NYHA functional class			0.912	0.14	0.03
NYHA I	23 (20.4)	23 (20.4)			
NYHA II	29 (25.7)	29 (25.7)			
NYHA III	36 (31.9)	32 (28.3)			
NYHA IV	25 (22.1)	29 (25.7)			
CCS grade			0.963	0.12	0.02
CCS 0	86 (76.1)	83 (73.5)			
CCS 1	14 (12.4)	17 (15.0)			
CCS 2	8 (7.1)	9 (8.0)			
CCS 3	3 (2.7)	2 (1.8)			
CCS 4	2 (1.8)	2 (1.8)			
EuroSCORE II	8.45 [5.85, 16.73]	8.62 [5.81, 12.81]	0.78	0.09	0.05
Body mass index, kg/m^2^	26.30 [24.21, 29.51]	26.47 [23.12, 28.91]	0.717	0.03	−0.10
Diabetes mellitus			0.958	0.19	0.02
Non-diabetics	94 (83.2)	94 (83.2)			
On diet control	3 (2.7)	2 (1.8)			
Oral medication	12 (10.6)	12 (10.6)			
Insulin	4 (3.5)	5 (4.4)			
Hypertension	60 (53.1)	56 (49.6)	0.69	0.04	0.07
Smoking			0.013	0.29	0.02
Never smoked	41 (36.3)	48 (42.5)			
Former smoker	71 (62.8)	56 (49.6)			
Current smoker	1 (0.9)	9 (8.0)			
Chronic kidney disease	16 (14.2)	12 (10.6)	0.545	0.17	−0.12
Chronic obstructive pulmonary disease	11 (9.7)	9 (8.0)	0.815	0.10	−0.06
Previous myocardial infarction	10 (8.8)	12 (10.6)	0.822	0.04	0.07
Previous cerebrovascular attack	16 (14.2)	17 (15.0)	1	0.03	0.02
Peripheral vascular disease	10 (8.8)	11 (9.7)	1	0.09	0.03
Preoperative atrial fibrillation	15 (13.3)	11 (9.7)	0.532	0.05	−0.10
Pulmonary hypertension	4 (3.5)	4 (3.5)	1	0.03	−0.07
Left ventricular ejection fraction			0.595	0.10	0.03
Poor < 30%	40 (35.4)	39 (34.5)			
Moderate 30–50%	1 (0.9)	0 (0.0)			
Good ≥ 50%	72 (63.7)	74 (65.5)			
Cardiogenic shock	6 (5.3)	3 (2.7)	0.496	0.06	0.14
Aortic valve explant: biological valve	85 (75.2)	87 (77.0)	0.876	0.29	0.04
Pre-op ventilation	3 (2.7)	4 (3.5)	1	0.13	0.05

CCS, Canadian Cardiovascular Society; NYHA: New York Heart Association.

Analysis of operative data (Table 3) revealed that the early reoperation group required significantly longer cardiopulmonary bypass times (median 135.00 min [IQR: 114.00, 185.00] vs 122.00 min [IQR: 98.00, 150.50], *P* = 0.005) and cross-clamp times (median 95.00 min [IQR: 77.00, 124.00] vs 88.00 min [IQR: 70.50, 104.00], *P* = 0.011) compared to the late group. The choice of prosthesis type was similar between groups, with a predominance of bioprosthetic valves (74.3% in the early group vs 75.2% in the late group, *P* = 1.000). Aortic valve sizes were also comparable (median 23.00 mm in both groups, *P* = 0.945). Post-operative outcomes showed that patients with reoperation ≤ 1 year had a longer median hospital stay (18.50 vs 14.00 days; *P* = 0.006). Re-exploration for bleeding (9.7% vs 7.1%; *P* = 0.632), cerebrovascular accidents (1.8% vs 3.5%, *P* = 0.679) and need for dialysis (9.7% vs 8.0%, *P* = 0.815) was similar (Table 4). While not reaching statistical significance, the mortality was higher in the early group (10.8% vs 6.2%, *P* = 0.338).

**Table 3:** Propensity-matched comparison of operative data

	Duration of reoperation for endocarditis ≤ 1 year (n = 113)	Duration of reoperation for endocarditis > 1 year (n = 113)	*P*
Aortic valve implant			
Bioprosthesis	84 (74.3)	85 (75.2)	1
Mechanical valve	29 (25.7)	28 (24.8)	
Aortic valve size	23.00 [21.00, 25.00]	23.00 [21.00, 23.00]	0.945
Aortic valve haemodynamic			0.969
Stenosis	24 (21.2)	25 (22.1)	
Regurgitation	77 (68.1)	77 (68.1)	
Mixed-stenosis + regurgitation	12 (10.6)	11 (9.7)	
Cardiopulmonary bypass time (min)	135.00 [114.00, 185.00]	122.00 [98.00, 150.50]	0.005
Cross clamp time (min)	95.00 [77.00, 124.00]	88.00 [70.50, 104.00]	0.011
Aortic valve explant (biological)	85 (75.2)	87 (77.0)	0.876

**Table 4:** Propensity-matched comparison of post-operative outcomes

	Duration of reoperation for endocarditis ≤ 1 year	Duration of reoperation for endocarditis > 1 year	*P*
	n = 113	n = 113	
Hospital length of stay (days)	18.50 [10.00, 34.00] *	14.00 [8.00, 22.00] *	0.006
Return to theatre for bleeding	11 (9.7%)	8 (7.1%)	0.632
Cerebrovascular accident	2 (1.8%)	4 (3.5%)	0.679
Dialysis	11 (9.7%)	9 (8.0%)	0.815
Post-op IABP use	1 (2.0%)	3 (7.3%)	0.486
Mortality	12 (10.8%)	7 (6.2%)	0.338

IABP, intra-aortic balloon pump.

*Median (Inter-quartile range)

**Figure 2:**
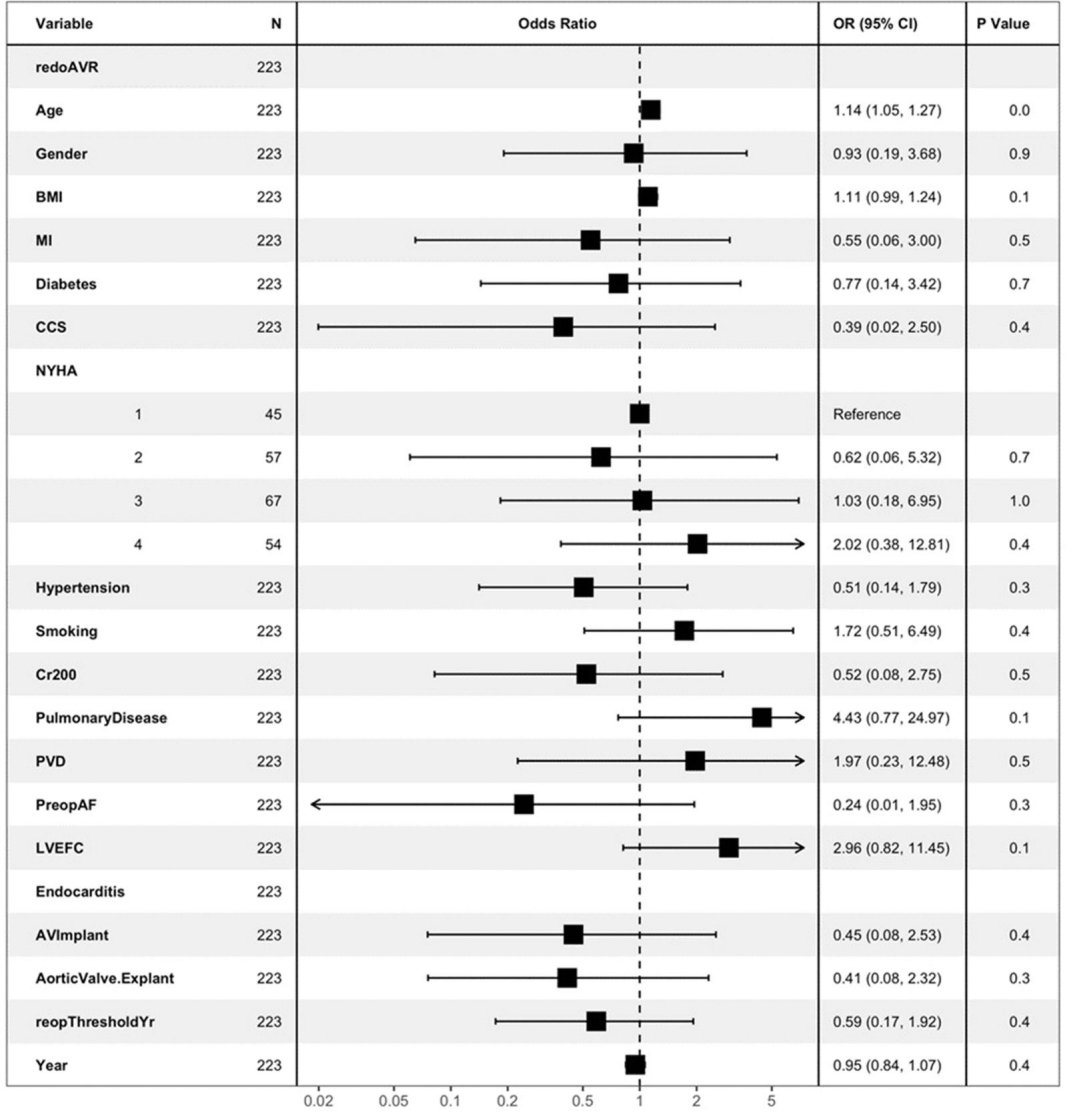
Forest plot for different risk factors for mortality in patients undergoing reoperation in patients with prosthetic aortic valve endocarditis.

The multivariable analysis identified several factors impacting mortality, with age emerging as the most significant predictor (OR: 1.14, 95% CI: 1.05–1.27, *P* = 0.0), indicating a 14% increase in risk per unit increase in age. This aligns with age being significant in 72.6% of bootstrap samples in the initial analysis. Pulmonary disease, significant in 36.2% of bootstrap samples, showed a notable though not statistically significant association (OR: 4.43, 95% CI: 0.77–24.97, *P* = 0.1). Similarly, body mass index (BMI), identified in 33.4% of bootstrap samples, did not reach statistical significance (OR: 1.11, 95% CI: 0.99–1.24) (Fig. 2).

The Lasso model obtained an AUC performance of 0.746 (Fig. S4) on the hold out dataset with the optimal lambda parameter of 0 selected in cross validation. The radial basis SVM model obtained a similar AUC of 0.755 (Fig. S5) on the hold out dataset with optimal parameter being C = 0.0020 and sigma = 0.21, respectively. However, it was found that reducing the number of variables were detrimental to the model prediction and hence all variables were included for prediction. This may also be explained by the inherent regularization capabilities of the models to prevent overfitting despite using more variables.

## DISCUSSION

The main findings of our study were that early reoperations (within 1 year of the initial surgery) were more prevalent in the initial period of the study but have since declined, with late reoperations (beyond 1 year) becoming increasingly prevalent in recent years. We also found that early prosthetic valve reoperations required longer cardiopulmonary bypass and cross-clamp times and had longer hospital stays compared to their late-reoperation counterparts. Although the mortality rate was numerically higher in the early reoperation group (10.8%) compared to the late group (6.2%), this difference did not reach statistical significance (*P* = 0.338). A multivariable analysis identified age as the most significant predictor of mortality, suggesting a 14% increased risk with each year of age.

Recent epidemiological studies have documented a significant upward trend in the incidence of PVE [11–13]. This was also observed in our study and the incidence of PVE seen in our study is similar to that reported from other the Danish and the Spanish registries [14, 15]. The increase in the incidence of PVE is driven by several interconnected factors. A significant contributor is the increasing number of elderly patients undergoing surgery, many of whom present with a higher burden of comorbidities and age-related immunosuppression, making them more susceptible to infection. Furthermore, advancements in diagnostic techniques, including echocardiography and nuclear imaging, combined with improved coding and data registration, have not only enhanced the detection and reporting of IE but also likely amplified its perceived incidence. Early PVE is predominantly considered a healthcare-associated infective endocarditis, and the microbiological milieu is different to late presenting endocarditis. It is also associated with greater incidence of heart failure and in-hospital mortality [16]. Despite an overall increase in the incidence of PVE, our study observed a gradual decline in early-onset PVE during the latter part of the study period. This trend may be attributed to significant advancements in surgical techniques, stringent infection control protocols, and the widespread adoption of evidence-based antibiotic regimens, which have collectively minimized healthcare-associated infections following AVR. Key measures likely contributing to this decline include preoperative screening and decolonization for *Staphylococcus aureus* nasal carriage, strict adherence to guideline-directed antibiotic therapy, and meticulous implementation of aseptic techniques. Furthermore, increased awareness and improved prophylactic strategies for patients with prosthetic heart valves, particularly those undergoing cardiac device implantation, have played an instrumental role in reducing the risk of early-onset PVE. While infection control measures are a plausible contributing factor for the improvement seen, this remains an area for further investigation. Meta-analyses comparing surgical aortic valve replacement (SAVR) and transcatheter aortic valve replacement (TAVR) have corroborated this early vulnerability period, with both procedures showing comparable temporal distributions of PVE within the first postoperative year [17]. This consistent early presentation highlights the critical importance of the immediate post-procedural period for infection risk and reinforces the need for targeted surveillance and preventive strategies during this vulnerable phase.

Evidence suggests that bioprosthetic valves carry a higher long-term risk of endocarditis compared to mechanical valves [18, 19]. This differential risk has been substantiated by comprehensive analyses from both the Swedish National Registry and the Society of Thoracic Surgeons' database [20, 21], with multivariate analyses showing a 1.6-fold increased risk after controlling for confounders [11]. This disparity may stem from the structural vulnerability of bioprosthetic valve leaflets to microbial colonization, unlike the pyrolytic carbon in mechanical valves, which resists infection. In our study, nearly 75% of valves associated with endocarditis were bioprosthetic. Some studies have identified a potential link between implanted valve type and mortality. However, many of these studies included patients undergoing concomitant procedures such as coronary artery bypass grafting, aortic surgery or multiple valve replacements. Additionally, they often considered both endocarditis and structural valve degeneration as indications for reoperation. In our study, we excluded patients requiring additional surgeries at the time of reoperation to maintain a focused analysis. Furthermore, we restricted the indication to endocarditis alone and used propensity matching to create two well-balanced patient cohorts. Our findings revealed no significant difference in mortality based on the implanted valve type, offering a more precise evaluation of outcomes specific to isolated reoperative AVR for endocarditis.

Our findings demonstrate a notable improvement in outcomes for patients with PVE, with an overall mortality rate of 8.4%. This represents a significant advancement compared to historical data from the UK Heart Valve Registry (1986–1996), which reported a mortality rate of 19.9% [22] and other international studies showing mortality rates ranging from 10 to 28% [5, 6, 23]. Particularly encouraging are the improved outcomes in early PVE cases (occurring within 12 months of initial valve surgery). While previous smaller studies documented mortality rates as high as 31% for early PVE compared to 9% for late cases, our large-scale nationwide analysis reveals more favourable outcomes: a 10.8% mortality rate for early PVE and 6.2% for late PVE. The robustness of our findings is supported by our comprehensive nationwide dataset, offering more generalizable results compared to earlier studies with limited sample sizes [16].

### Limitations

Several limitations of our study warrant consideration. First, as with all retrospective analyses, our study is subject to inherent selection bias and confounding factors. While propensity matching was employed to mitigate these issues, residual confounding cannot be entirely excluded. Second, the 24-year study period (1996–2019) spans significant advancements in surgical techniques, medical management and antimicrobial strategies, which may have independently influenced patient outcomes beyond the factors analysed. The NICOR database, while comprehensive, may not capture all relevant variables that could influence PVE outcomes, such as specific microbial pathogens, antibiotic protocols or detailed surgical technical aspects. While the cases were classified as PVE in the registry, the absence of microbiological confirmation is an important limitation in ensuring diagnostic accuracy. The registry’s scope is limited to in-hospital outcomes, which precludes the assessment of long-term outcomes, including long-term mortality. Furthermore, key data such as the duration of antibiotic therapy and the interval between infection diagnosis and surgery were not available. The study’s inability to assess PVE patients managed with medical management alone is also a significant limitation and prevents a comprehensive comparison between surgical and non-surgical management strategies. Lastly, the analysis was limited to patients undergoing reoperative surgery for PVE, and therefore, the findings may not reflect the broader population of PVE patients, particularly those managed non-surgically or deemed unsuitable for reoperation. Finally, we excluded patients who required root replacements or any concomitant procedure due to presence of root abscesses or had more extensive destruction. As this is registry data, no detailed echo findings were available to us for these patients. These limitations should be considered when interpreting the study results.

## CONCLUSION

This large-scale, nationwide analysis of reoperative aortic valve PVE cases reveals several important findings. The temporal shift from predominantly early to late reoperations over the study period suggests evolving patterns in PVE presentation and management. Age remains the most significant predictor of mortality. While early reoperations were associated with more complex procedures and prolonged hospital stays, we did not observe statistically significant differences in mortality between early and late reoperations. Future research should focus on development of risk-stratification tools incorporating ctiming-specific factors and long-term survival data.

## Supplementary Material

ivaf096_Supplementary_Data

## Data Availability

The data underlying this article were provided by National Institute of Cardiovascular Outcomes Research (NICOR). Data can be shared on request to the corresponding author subject to permission from NICOR.

## Notes

Presented at the 38th EACTS Annual Meeting, Lisbon, Portugal, October 9–12, 2024.

## ABBREVIATIONS

BMI: Body mass index
CCS: Canadian Cardiovascular Society
CI: Confidence interval
CVA: Cerebrovascular accident
HAIs: Healthcare-associated infections
HCRW: Health and Care Research Wales
HRA: Health Research Authority
IABP: Intra-aortic balloon pump
IRAS: Integrated research application system
IQR: Interquartile range
LVEF: Left ventricular ejection fraction
MI: Myocardial infarction
NACSA: National Adult Cardiac Surgery Audit
NICOR: National Institute of Cardiovascular Outcomes Research
NYHA: New York Heart Association
OR: Odds ratio
PVD: Peripheral vascular disease
PVE: Prosthetic valve endocarditis
UK: United Kingdom
