# Supplementary material for: Reoperative surgery for early- and late-onset prosthetic valve endocarditis: temporal trends and outcomes
Source: Interdiscip Cardiovasc Thorac Surg. 2025 Apr 11;40(4):ivaf096. doi: 10.1093/icvts/ivaf096 (PMC12036968; doi:10.1093/icvts/ivaf096)
Supplement: ivaf096_Supplementary_Data [file ivaf096_supplementary_data.zip › 2025-03-03-Supplementary file.docx]

| Supplementary Table 1: Covariates included in the comprehensive logistic regression model |
| --- |
| Age |
| Gender |
| Body mass index (BMI) |
| New York Heart Association (NYHA) functional class (categorized as I-IV) |
| Canadian Cardiovascular Society (CCS) angina class |
| Diabetes Mellitus |
| Hypertension |
| Smoking History |
| History of myocardial infarction (MI) |
| Chronic kidney disease (taken as creatinine >200 mg/dl) |
| Chronic obstructive pulmonary disease |
| Prior cerebrovascular accident (CVA) |
| Peripheral vascular disease (PVD) |
| Left ventricular ejection fraction (LVEFC) |
| Preoperative atrial fibrillation (preopaf) |
| Aortic valve haemodynamic |
| Aortic valve implant type(avimplant), |
| Aortic valve explant type |
| Year of the operation |

| Supplementary Table 2: Covariates included in the comprehensive logistic regression model |
| --- |
| Age |
| Gender |
| Body mass index (BMI) |
| New York Heart Association (NYHA) functional class (categorized as I-IV) |
| Canadian Cardiovascular Society (CCS) angina class |
| Diabetes Mellitus |
| Hypertension |
| Smoking History |
| Preoperative atrial fibrillation (preopaf) |
| History of myocardial infarction (MI) |
| Chronic kidney disease (taken as creatinine >200 mg/dl) |
| Chronic obstructive pulmonary disease |
| Prior cerebrovascular accident (CVA) |
| Peripheral vascular disease (PVD) |
| Left ventricular ejection fraction (LVEF) |
| Aortic valve haemodynamic |
| Aortic valve implant type(avimplant), |
| Aortic valve explant type |
| Year of the operation |
| Timing of presentation (early≤1 year vs. Late>1 year reoperation) |


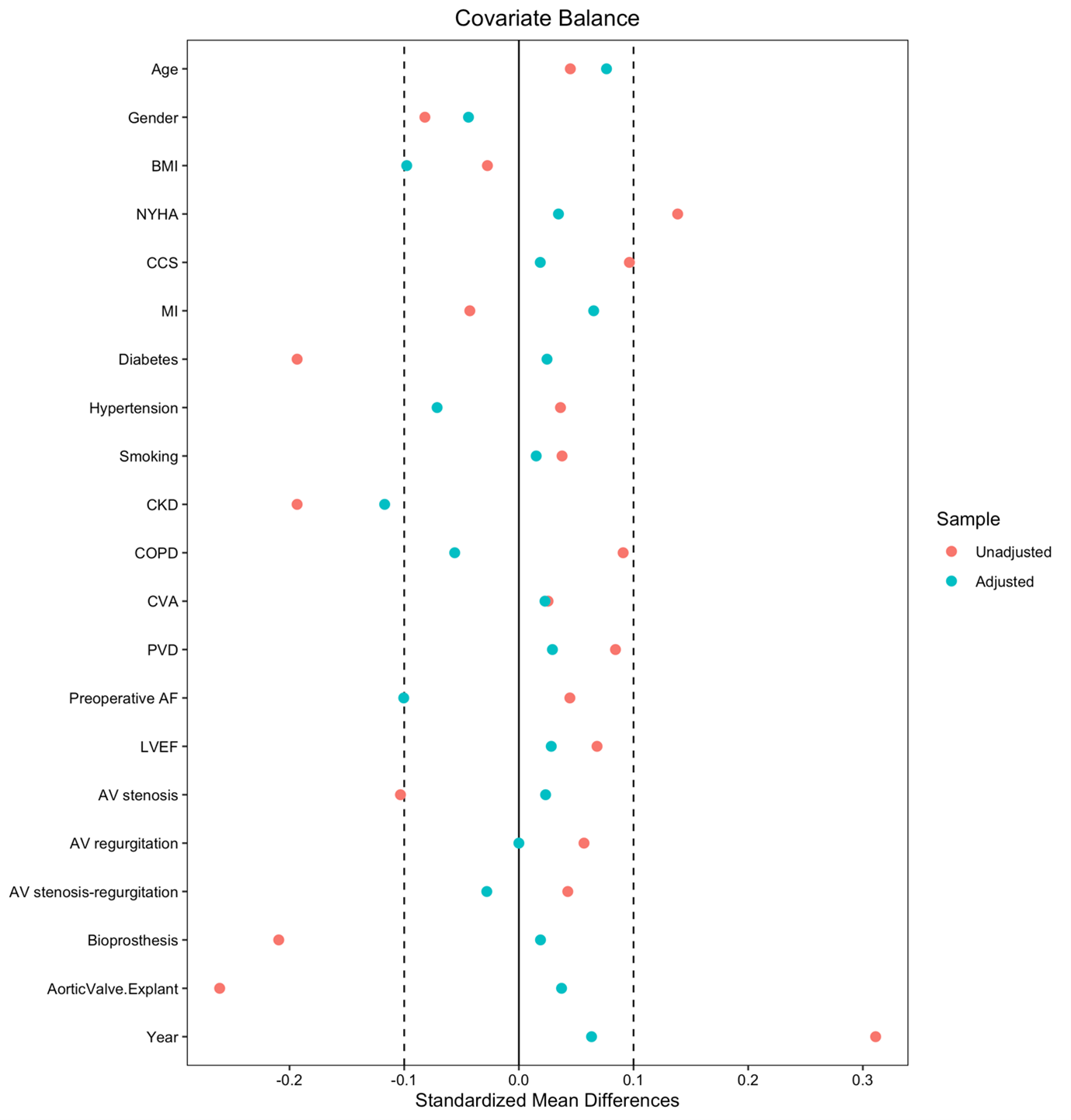


Supplementary Figure S1: Balance of propensity matching of patients in early (≤1 year) versus late (>1 year) reoperation group.


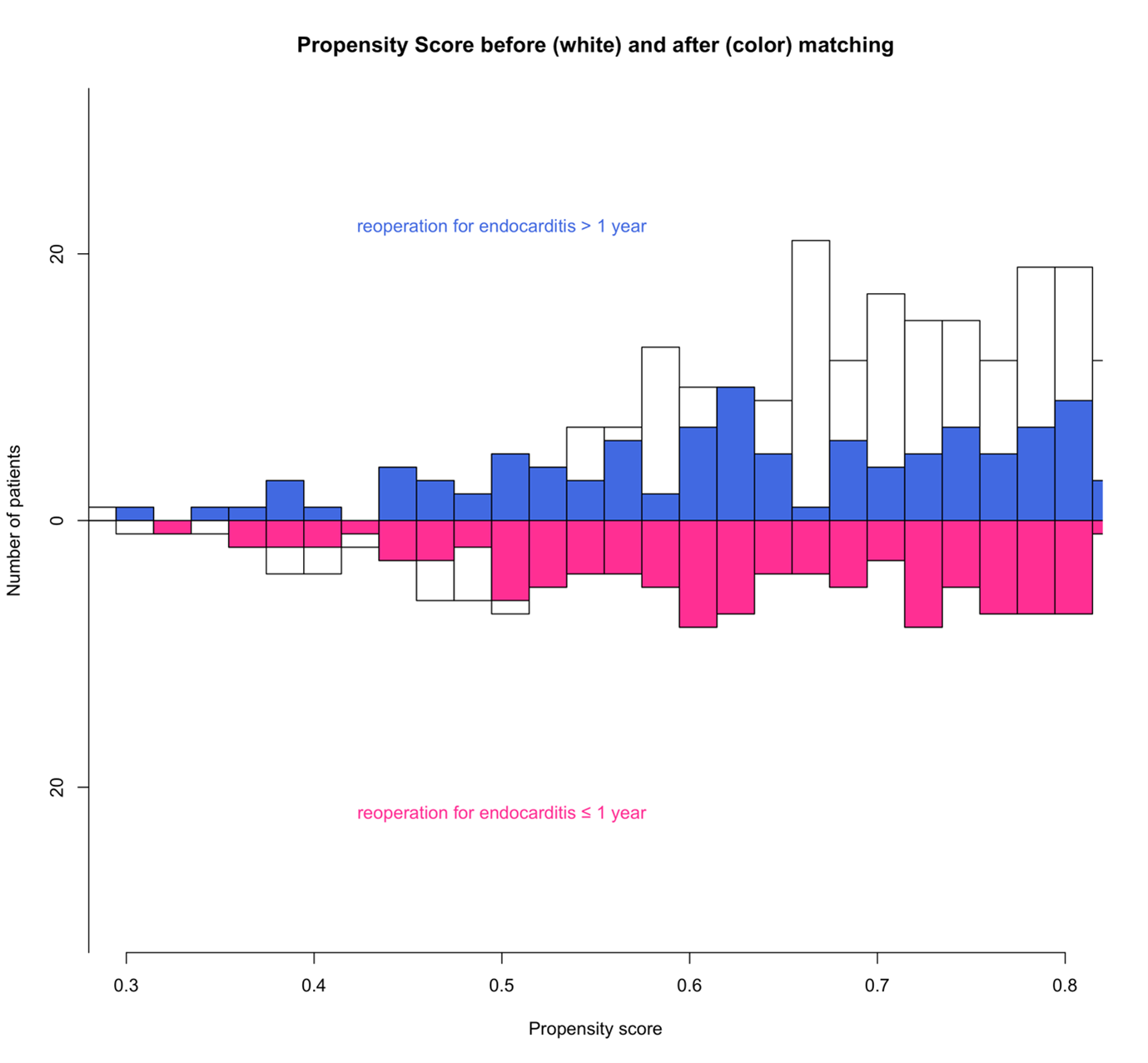


Supplementary Figure S2: Propensity scores before (white) and after matching (coloured) to assess efficiency of propensity matching.


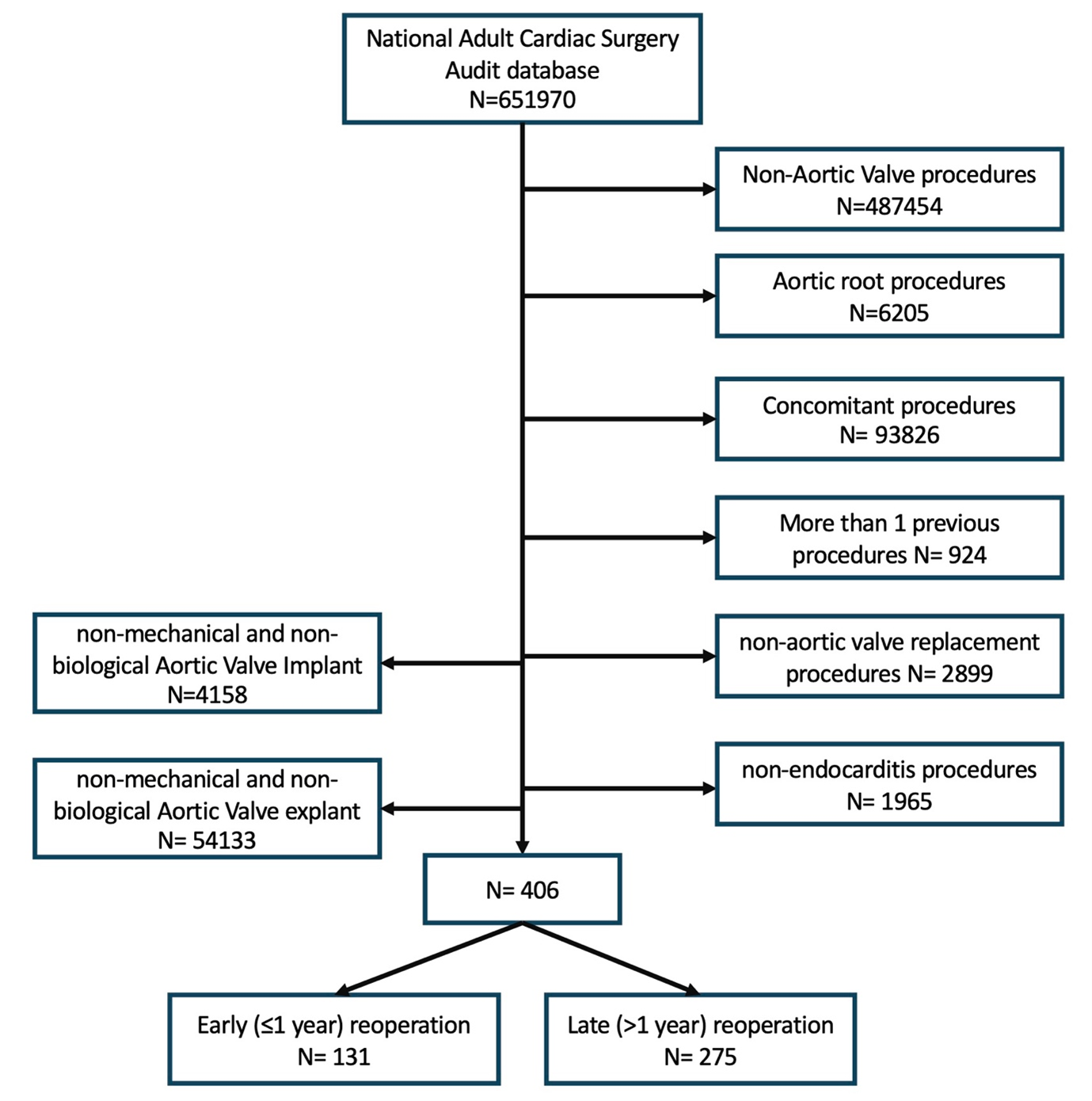

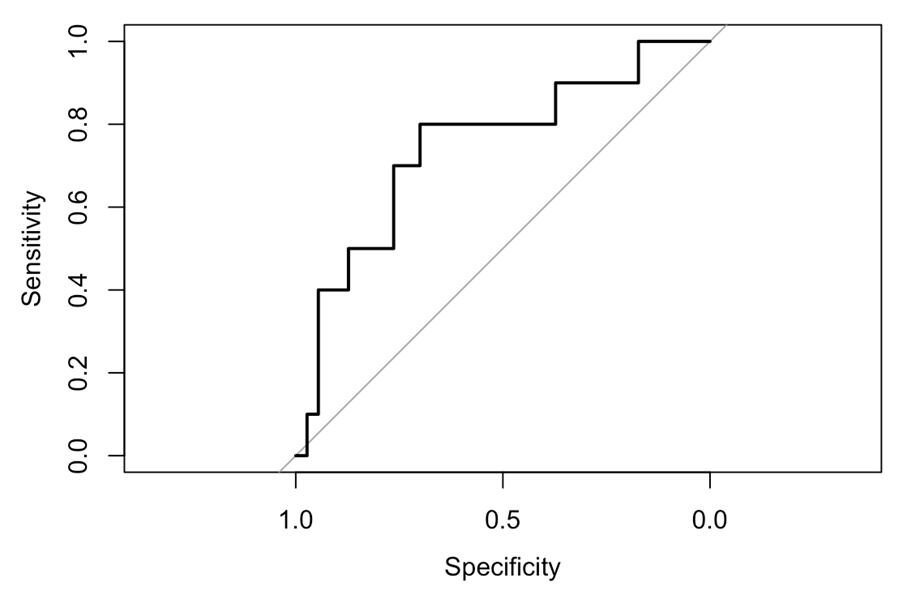


Supplementary Figure S3: CONSORT (Consolidated Standards of Reporting Trials) diagram of the study.

Supplementary Figure S4 ROC curve plot for the Lasso model performance on the hold out dataset using lasso; AUC = 0.746.


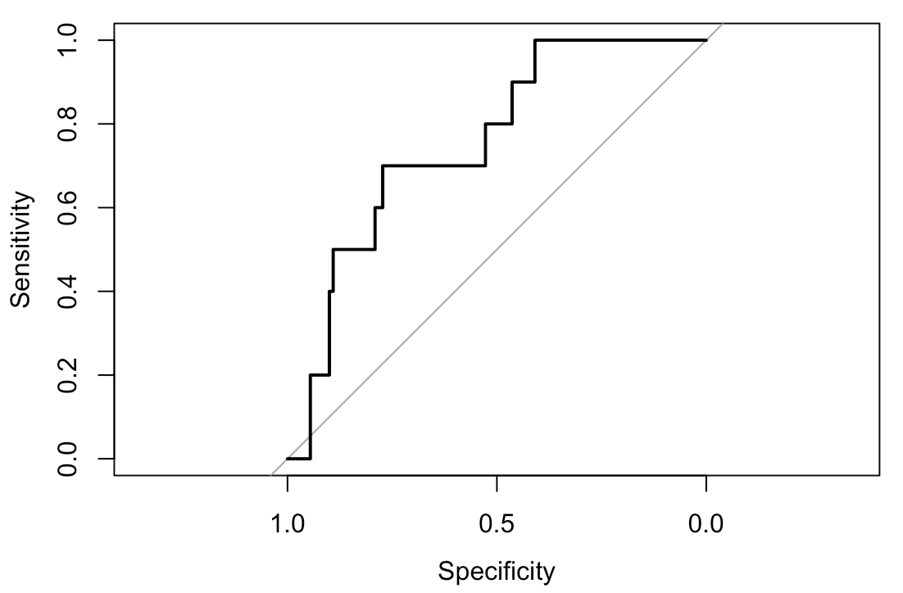


Supplementary Figure S5 ROC curve plot for the Lasso model performance on the hold out dataset using radial basis SVM; AUC = 0.755.
