# Supplementary figures and images for: Reoperative surgery for early- and late-onset prosthetic valve endocarditis: temporal trends and outcomes
Source: Interdiscip Cardiovasc Thorac Surg. 2025 Apr 11;40(4):ivaf096. doi: 10.1093/icvts/ivaf096 (PMC12036968; doi:10.1093/icvts/ivaf096)

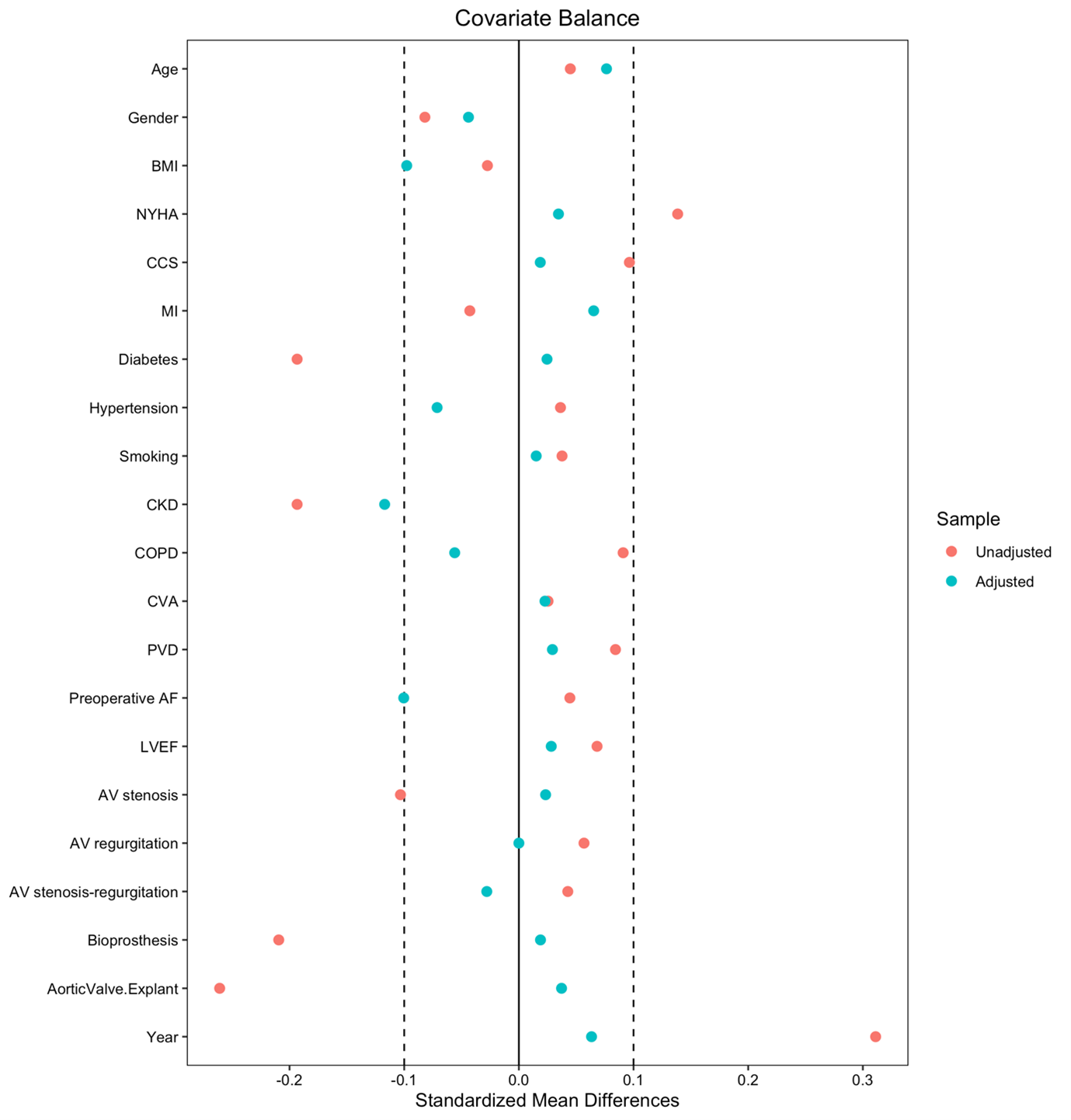

Supplement: ivaf096_Supplementary_Data [file ivaf096_supplementary_data.zip › Supplementary Figure S1.tif]

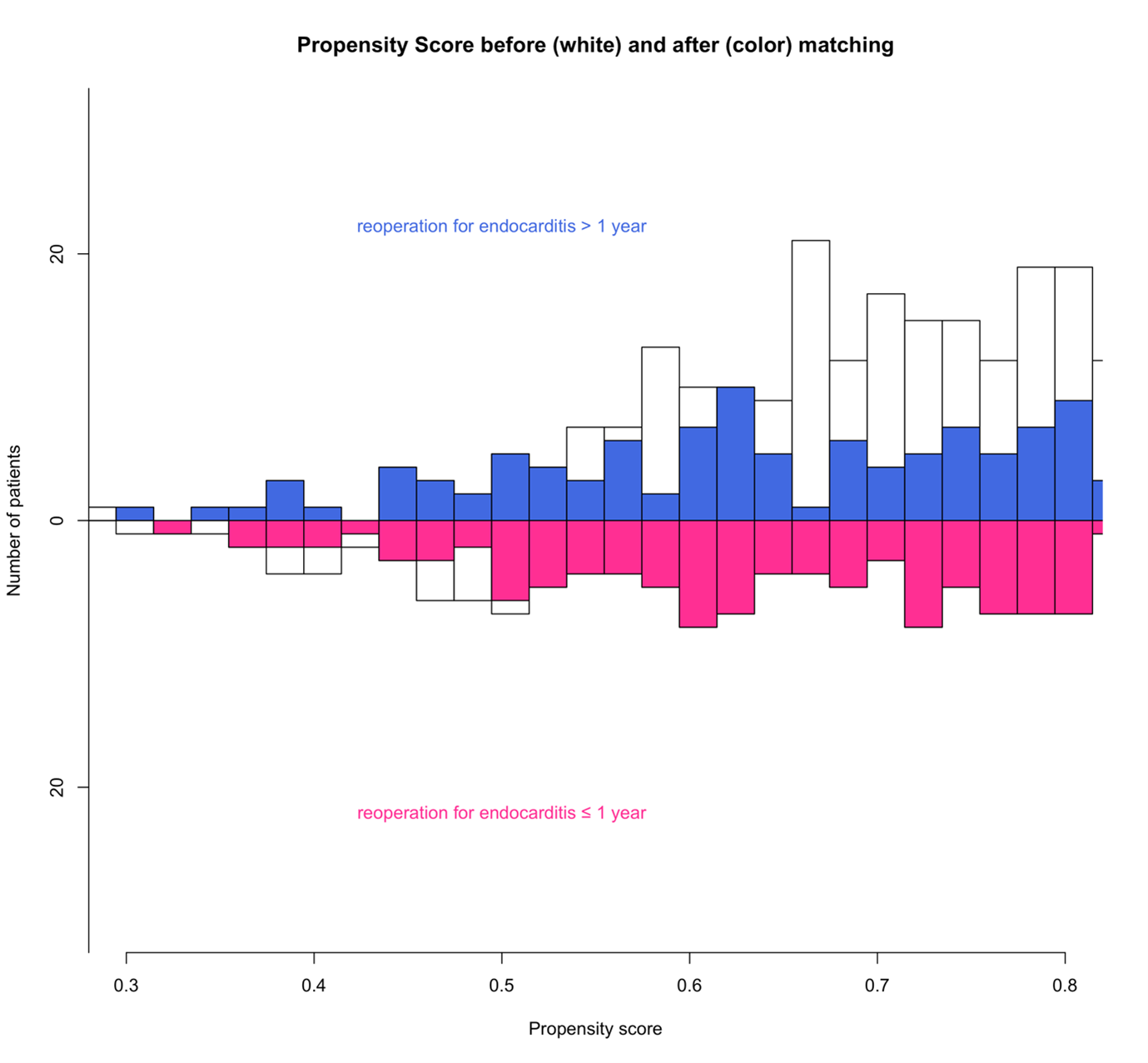

Supplement: ivaf096_Supplementary_Data [file ivaf096_supplementary_data.zip › Supplementary Figure S2.tif]

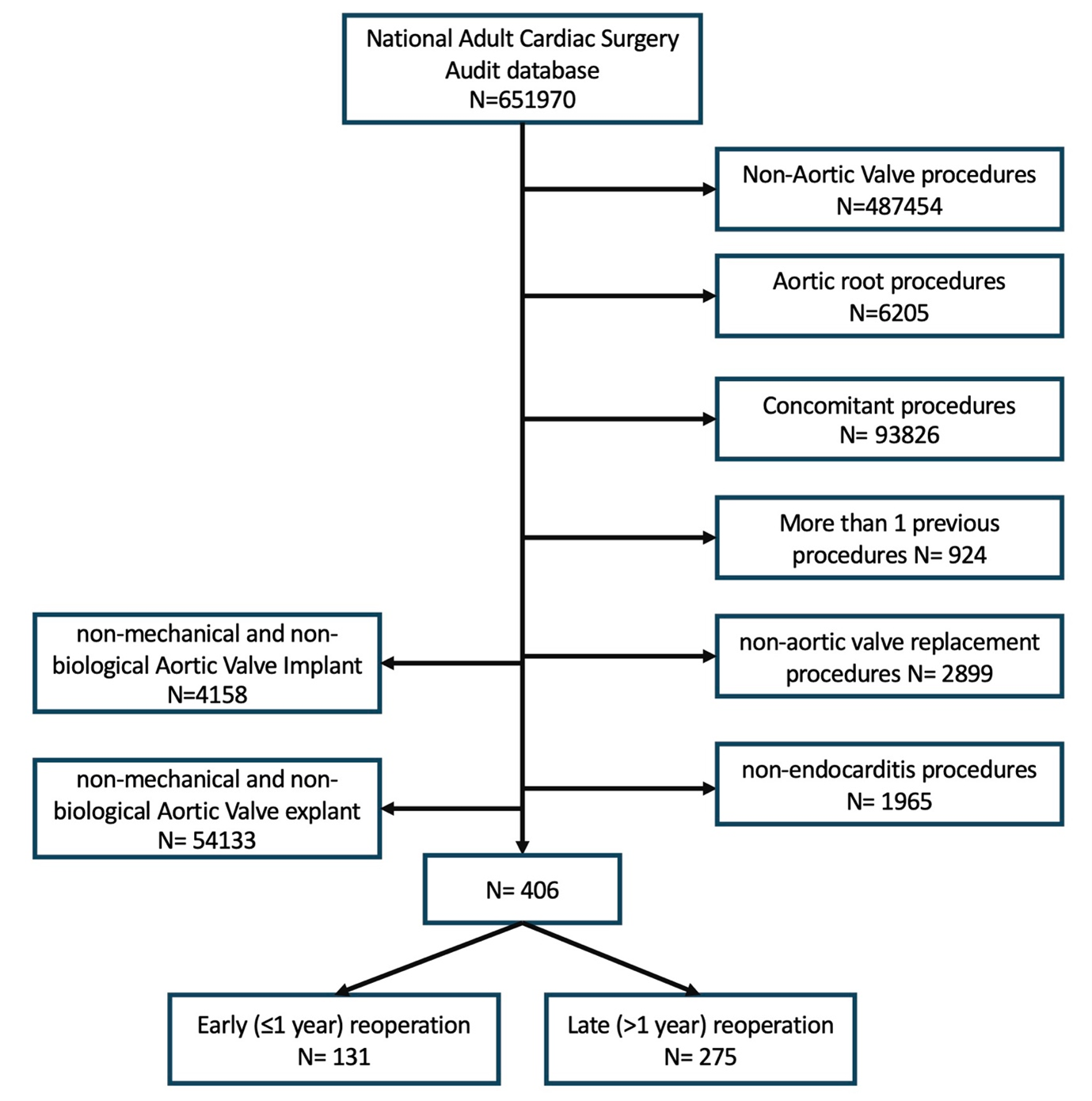

Supplement: ivaf096_Supplementary_Data [file ivaf096_supplementary_data.zip › Supplementary Figure S3.tif]
